# Supplementary material for: From sequence to enzyme mechanism using multi-label machine learning
Source: BMC Bioinformatics. 2014 May 19;15:150. doi: 10.1186/1471-2105-15-150 (PMC4229970; doi:10.1186/1471-2105-15-150)
Supplement: Additional file 2 — Java code of ml2db. Additional file ml2db_code.tar.gz contains the Java source code to run the multi-label machine learning experiments and save the results to database. The code’s Javadoc is included. [file 1471-2105-15-150-S2.zip › additional file 2/ml2db/ml2db/doc/mulan/data/UnconditionalChiSquareIdentifier.html]

UnconditionalChiSquareIdentifier


JavaScript is disabled on your browser.


- Overview
- Package
- Class
- Use
- Tree
- Deprecated
- Index
- Help

- Prev Class
- Next Class

- Frames
- No Frames

- All Classes

- Summary:
- Nested |
- Field |
- Constr |
- Method

- Detail:
- Field |
- Constr |
- Method


mulan.data

## Class UnconditionalChiSquareIdentifier

- java.lang.Object
- - mulan.data.UnconditionalChiSquareIdentifier

- All Implemented Interfaces:
  :   java.io.Serializable, LabelPairsDependenceIdentifier

  ---

    

  ```
  public class UnconditionalChiSquareIdentifier
  extends java.lang.Object
  implements LabelPairsDependenceIdentifier, java.io.Serializable
  ```

  A class for identification of unconditional dependence between each pair of labels using Chi Square Test For Independence.
  The chi-square test for independence is applied to the number of instances for each possible combination of two categories.

  Version:
  :   30.11.2010

  Author:
  :   Lena Chekina (lenat@bgu.ac.il)

  See Also:
  :   Serialized Form

- - ### Constructor Summary

    Constructors

    Constructor and Description

    `UnconditionalChiSquareIdentifier()`
  - ### Method Summary

    Methods

    | Modifier and Type | Method and Description |
    | --- | --- |
    | `LabelsPair[]` | `calculateDependence(MultiLabelInstances mlInstances)` Calculates Chi Square values for each pair of labels. |
    | `double` | `getCriticalValue()` Returns a critical value |
    | `void` | `setCriticalValue(double criticalValue)` |

    - ### Methods inherited from class java.lang.Object

      `equals, getClass, hashCode, notify, notifyAll, toString, wait, wait, wait`

- - ### Constructor Detail


    - #### UnconditionalChiSquareIdentifier

      ```
      public UnconditionalChiSquareIdentifier()
      ```
  - ### Method Detail


    - #### calculateDependence

      ```
      public LabelsPair[] calculateDependence(MultiLabelInstances mlInstances)
      ```

      Calculates Chi Square values for each pair of labels. It uses Phi correlation value calculated in `Statistics` as follows: ChiSquareValue = PhiValue^2 \* NumberOfInstances in the data set.

      **Specified by:**
      :   `calculateDependence` in interface `LabelPairsDependenceIdentifier`

      Parameters:
      :   `mlInstances` - multilabel data set

      Returns:
      :   an array of label pairs sorted in descending order of the ChiSquare value


    - #### setCriticalValue

      ```
      public void setCriticalValue(double criticalValue)
      ```

      Parameters:
      :   `criticalValue` -


    - #### getCriticalValue

      ```
      public double getCriticalValue()
      ```

      **Description copied from interface: `LabelPairsDependenceIdentifier`**

      Returns a critical value

      **Specified by:**
      :   `getCriticalValue` in interface `LabelPairsDependenceIdentifier`

      Returns:
      :   critical value


- Overview
- Package
- Class
- Use
- Tree
- Deprecated
- Index
- Help

- Prev Class
- Next Class

- Frames
- No Frames

- All Classes

- Summary:
- Nested |
- Field |
- Constr |
- Method

- Detail:
- Field |
- Constr |
- Method
